# Antibody Response against Severe Acute Respiratory Syndrome Coronavirus 2 Messenger Ribonucleic Acid Vaccines in Infected Individuals: A Systematic Review

**DOI:** 10.21315/mjms2023.30.4.2

**Published:** 2023-08-24

**Authors:** Madihah Roslan, Farah Ratulfazira Mohd Nisfu, Mohd Hafiz Arzmi, Ridhwan Abdul Wahab, Norafiza Zainuddin

**Affiliations:** 1 Department of Biomedical Science, Kulliyyah of Allied Health Sciences, International Islamic University Malaysia, Pahang, Malaysia; 2 Department of Fundamental Dental and Medical Sciences, Kulliyyah of Dentistry, International Islamic University Malaysia, Pahang, Malaysia; 3 International Medical School, Management and Science University, Selangor, Malaysia

**Keywords:** COVID-19, SARS-CoV-2, mRNA vaccine, vaccine, antibodies, antibody response

## Abstract

Individuals with a history of coronavirus disease 2019 (COVID-19) exhibit memory immunity acquired during natural infection. However, a decline in immunity after infection renders these individuals vulnerable to re-infection, in addition to a higher risk of infection with new variants. This systematic review examined related studies to elucidate the antibody response in these infected individuals after messenger ribonucleic acid (mRNA) vaccination. Hence, the focus of this review was to ascertain differences in the concentration of binding and neutralising antibodies of previously infected individuals in comparison to those of infection-naïve individuals after administration of two doses of mRNA vaccination through available case-control and cohort studies. Positive reverse transcriptase-polymerase chain reaction (RT-PCR) test or detectable anti-severe acute respiratory syndrome coronavirus 2 (SARS-CoV-2) antibodies at the baseline in included studies showed categorisation of infected and uninfected individuals. This review utilised three online databases: PubMed, Scopus and Cochrane with the following keywords: (COVID-19 OR ‘Coronavirus Disease 2019’ OR SARS-CoV-2) AND Immun* AND (Pfizer OR BioNTech OR BNT162b2 OR Comirnaty OR Moderna OR mRNA-1273) from January 2019 to July 2021. Following the Preferred Reporting Items for Systematic Review and Meta-Analysis Protocol (PRISMA-P) 2020 guidelines and assessment based on the Crowe Critical Appraisal Tool (CCAT), we included 13 related qualified papers of observational studies discerning the binding and neutralising antibody concentrations of infected and uninfected individuals after administration of mRNA vaccines, such as the BNT162b2 and mRNA-1273 vaccine. The mRNA vaccines induced robust binding and neutralising antibody responses in both groups. However, infected individuals showed induction of higher antibody responses in a shorter time compared to uninfected individuals. Hence, a single dose of mRNA vaccination for infected individuals may be sufficient to reach the same level of antibody concentration as that observed in uninfected individuals after receiving two doses of vaccination.

## Introduction

Coronavirus disease 2019 (COVID-19) is a communicable disease induced by a novel coronavirus strain, severe acute respiratory syndrome coronavirus 2 (SARS-CoV-2). The rapid spread of COVID-19 infection led to approximately two and a half years of the pandemic. A countless number of daily cases and deaths attributed to this virus have been reported worldwide. Apart from a deep economic recession and a huge reduction in employment due to continuous lockdown, the healthcare system of many countries is on the brink of collapse. Daily cases lead to high hospitalisation rates, which consequently lead to a shortage of beds and staff for proper treatment services. Efforts have been made to manage the contagious spread of the virus. Control measures, such as physical distancing, masks, appropriate hygiene measures and temperature checks, have been adopted to disrupt the infection chain to return to pre-pandemic normalcy. Vaccination is a possible method for controlling and overcoming these obstacles (1). Vaccination cannot only prevent severe infection but can also efficiently intercept viral transmission, leading to herd immunity. Many types of vaccines have been developed to combat the infection spread, including the novel messenger ribonucleic acid (mRNA) technology-based vaccine.

Currently, two mRNA vaccines, BNT162b2 and mRNA-1273, have been authorised by the United States Food and Drug Administration (USFDA) for COVID-19 (2, 3). Both vaccines are designed to express genetic sequences that translated into a full-length spike glycoprotein (4) with antigenic alteration of two proline mutations: i) K986P and ii) V987P, which are often abbreviated as S-2P (5, 6). Multiple clinical trials have demonstrated the efficacy, safety and immunogenicity in healthy adults for this type of vaccine (7–13). Individuals with a history of COVID-19 demonstrate immunity against the virus acquired due to natural infection. However, this immunity is very likely to wane over time (14, 15). Hence, through available case-control and cohort studies, this review focuses on ascertaining differences in the concentration of binding and neutralising antibodies of these infected individuals in comparison to those of infection-naïve individuals after administration of two doses of mRNA vaccination.

The review question was constructed based on the PICO formulation; i.e. P = the population of the studies, I = intervention or exposure, C = comparison of intervention or exposure and O = outcome of the interest (18). This formulation was suggested by the Cochrane Handbook for Systematic Review for Interventions (19) as a model for developing review questions and search terms. Hence, the formulated questions for this review were “Will antibody responses be induced in COVID-19-infected individuals after receiving mRNA vaccines?” and “Will the level of antibody titres in individuals previously infected with COVID-19 who received the mRNA vaccines be similar to those of uninfected individuals?” The findings from this systematic review may assist future researchers in understanding the biological reactions of mRNA vaccines. This study may help in spreading awareness in the community on the importance of vaccination to promote long-lasting immunity against SARS-CoV-2.

## Methods

**Figure 1:**
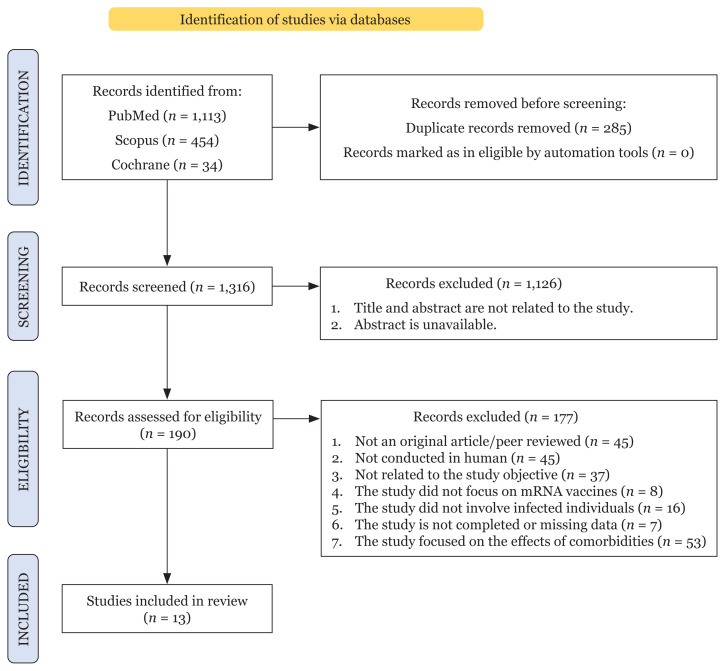
The PRISMA-P 2020 flow chart

The Preferred Reporting Items for Systematic Review and Meta-Analysis Protocol (PRISMA-P) 2020 guidelines were followed (16) (Figure 1). These guidelines aim to improve the quality of systematic review protocols, similar to the impact of other reporting guidelines (17–19).

### Search Strategy

This systematic review concentrated on available studies that demonstrated the immune responses of mRNA vaccines for COVID-19 by employing three different online databases, namely, PubMed, Scopus and Cochrane. PRISMA-P (16) 2020 guidelines were used for the selection procedure of related articles. This study was systematically performed by inserting the keywords (COVID-19 OR ‘Coronavirus Disease 2019’ OR SARS-CoV-2) AND Immun* AND (Pfizer OR BioNTech OR BNT162b2 OR Comirnaty OR Moderna OR mRNA-1273) identically in the aforementioned databases.

### Inclusion and Exclusion Criteria

The inclusion criteria for this systematic review involved original articles with an observational study design. In addition, the language was defined as English with a publication period of January 2019 to July 2021. The articles were peer-reviewed. Studies focusing on the binding and neutralising antibody response of mRNA vaccines, such as BNT162b2 and mRNA-1273 vaccines, were included. In addition, articles focusing on individuals infected with COVID-19 were strictly counted. The terms infected and uninfected/naïve-infected are used in this study to elucidate differences in the COVID-19 infection status. Individuals with a history of SARS-CoV-2 infection and recovery were termed ‘infected individuals’. The history of infection was confirmed with a positive molecular diagnosis based on reverse transcriptase-polymerase chain reaction (RT-PCR) and/or positive serological diagnostic tests or with blood tests conducted during the pre-vaccination stage that showed measurable anti-SARS-CoV-2 antibodies. Individuals with a negative RT-PCR diagnosis, and/or a negative serological diagnosis and non-measurable anti-SARS-CoV-2 antibodies were categorised in the uninfected group and termed ‘infection-naïve’ (i.e. those who were not infected with SARS-CoV-2 or those who had no history of COVID-19 infection). Other articles, such as those with the absence of abstract and full text, articles involving studies examining non-human subjects, those involving examination of vaccines for variants of concern (VOC) and variants of interest (VOI), and papers focusing on the response of socio-demographic factors were excluded.

### Inter-rater Reliability

In a systematic review, inter-rater reliability can be used to evaluate the agreement between reviewers during data extraction. Two reviewers screened the studies for inclusion in this systematic review based on the inclusion and exclusion criteria, with the main reviewer screening the records for inclusion and the other verifying all decisions. In this review, inter-rater reliability was measured using percent agreement and Cohen’s kappa coefficient. Percent agreement is an amount of the actually observed agreement. Cohen’s kappa coefficient score was calculated using the combination of percent agreement and chance agreement formulas, whereby both formulas require the scores rated by each author for the suitability of all included articles. The interpretation of Cohen’s kappa results was conducted based on McHugh (20), whereby Cohen’s kappa scores above 0.80 indicate a strong level of agreement among the raters, and a high confidence should be placed in the study results. According to McHugh (20), a negative Cohen’s kappa score represents an agreement worse than expected or disagreement. Low negative values (0 to −0.10) may generally be interpreted as ‘no agreement’. A large negative Cohen’s kappa value represents significant disagreement among raters. Data collected under conditions of such disagreement among raters are unlikely to represent the facts of the situation (whether research or clinical data) with any meaningful degree of accuracy. Such findings require either rater retraining or instrument redesign.

### Quality Assessment

The Crowe Critical Appraisal Tool (CCAT) is used to assess the quality and integrity of the articles included and to avoid any biases that are present in the study (21). It is a requirement to use the CCAT form along with the CCAT user guide co-operatively for quality assessment of the articles. The resulting checklist comprised eight categories: i) preliminaries; ii) introduction; iii) design; iv) sampling; v) data collection; vi) ethical matters; vii) results and viii) discussion, with a 6-point scale (0, 1, 2, 3, 4 and 5) for each category. The total of these points was divided by 40 to obtain the total percentage scores of the articles. The percentage was then classified into three groups: high (≥ 75%), moderate (51%–74%) or low (≤ 50%) quality of articles (22, 23). Only articles with high and moderate quality were accepted for inclusion in this study.

## Results

### Study Selection

The keywords inserted in the search strings of three different databases resulted in a total of 1,601 articles (as of 8 July 2021). Amongst them, only 1,316 articles were considered relevant for the screening process owing to the duplication of 285 articles. Additionally, during the screening process of the title and abstract 1,126 articles are excluded as they were not within the scope of this review and/or the abstracts were unavailable. Then, the assessment of eligibility for the remaining 190 titles of articles was conducted based on the inclusion and exclusion criteria. Of the remaining 190 articles, the final step of study selection resulted in 13 articles that were included in this systematic review. The percentage of agreement in the selection of articles was 92.3%, and Cohen’s kappa score of inter-rater reliability was 0.806. The summary of this study selection performed using the PRISMA-P 2020 guidelines is presented in Figure 1.

### Study Characteristics

The study design for included articles were case-control (23, 24, 31, 32, 34, 35) and cohort (25–30, 33) studies. All articles focused on the level of humoral immune response of mRNA vaccines at different time points of vaccination in previously infected and infection-naïve healthcare workers. Furthermore, specifically, immunoglobulin G (IgG) antibody response was observed in all studies against the receptor-binding domain (RBD) or spike glycoprotein of SARS-CoV-2. Correspondingly, all studies examined either the binding or neutralising antibody concentrations. Vaccination time points in the studies evaluated the antibody concentration at the baseline (pre-vaccination) and after the first and second immunisation by analysis of blood plasma samples of participants. Most studies used different serological assays to measure the antibody titres. The overview of the characteristics of included articles described above is provided in Table 1.

The median values of binding and neutralising antibody concentrations in infected and uninfected individuals retrieved from the articles are displayed in Table 2. Different units are presented to express the antibody concentrations of both binding and neutralising antibodies as different immunoassays were used in the studies.

### Level of Antibody Response toward mRNA Vaccines in Infected Individuals

Table 3 illustrates the percentage increase in the concentration of binding and neutralising antibodies based on the vaccination time points in formerly infected individuals. The interpretation of the concentration changes is also included in this table, which can be translated into either a robust increase or a minor increase or decrease. For the baseline value, detectable or undetectable interpretation was also shown as no calculation of changes was needed. Based on the articles, an increase of more than 88% in the concentration was considered a robust increase, while an increase of less than 57% was considered a minor increase. In addition, an increase below 10% in concentration was defined as not significant (*P* > 0.999). An increase between 57% and 88% was considered as an increase only. No specific values were mentioned by the authors, indicating the strength of the concentration changes. Hence, these percentages of increase were determined by compiling the concentration data from the included articles.

The mRNA vaccines examined in all studies showed the detectability of IgG antibodies during the pre-vaccination assessment (at baseline) in previously infected individuals. In 11 studies (23, 25–33) and (35) that investigated binding antibody responses, robust and rapid increases were observed after administration of the first dose except for the study published by Ebinger et al. (26), which showed only a minor increase in response when compared to the baseline values. Meanwhile, following the booster dose, most of the studies (23, 27, 28, 31–33, 35) noticed further small increases in binding antibody titres. However, one article for each observation of robust increase (35) and non-additional antibody concentration (30) after the second immunisation were perceived when it is analogised to the first dose responses.

All studies that examined neutralising antibody responses (23, 24, 26, 28) noticed the detectability of antibodies specialised for SARS-CoV-2 antigen at the baseline. In addition, these five studies also observed steep increases in neutralising titres after the primary dose. Correspondingly, following the recall dose, three articles (23, 26, 28) examined small increments in responses, while Modenese et al. (34) observed robust increases.

### Comparison of Antibodies Response towards mRNA Vaccines in Infected and Uninfected Individuals

A comparison of concentrations between infected and uninfected individuals (based on the data presented in Table 2) is presented in Table 4. All studies showed higher binding antibody response in previously infected individuals at all vaccination time points (i.e. at baseline and after the first and second doses). However, a few studies (25, 26, 28) were the exception; the results demonstrated only higher responses at baseline and after the first dose. Meanwhile, Kontou et al. (35) detected higher titres following the primary and secondary doses only because pre-vaccination assessments were not conducted. Notably, seven studies (26, 27) and (29–33) demonstrated higher binding antibody titres in previously infected individuals after the first dose when compared to the titres in infection-naïve individuals after receiving the second dose. Furthermore, lower binding antibody responses in seropositive individuals than those in seronegative individuals after the administration of the booster dose were observed by Goel et al. (28).

The results of all related studies (23, 26, 28) examining neutralising responses exhibited higher antibody responses in infected individuals at all vaccination time points. The exceptions involved the study conducted by Vicenti et al. (24) that only measured a higher concentration at the baseline and after the first dose and the study conducted by Modenese et al. (34) that only observed higher neutralisation responses at the baseline and after the booster dose. Furthermore, Vicenti et al. (24) and Goel et al. (28) presented remarkable data that showed higher titres after the primary dose in naturally infected individuals when compared to those observed after the recall dose in infected-naïve individuals.

### Risk of Bias Assessment

Table 4 shows the result of the bias risk assessment and Table 5 displays the final results of the assessments that correspondingly signify the quality of the articles. Based on the tables, 8 of the 14 articles (23, 25–28, 30, 33, 34) expressed high percentage scores (> 75%) of quality assessment while the other five articles (24, 29, 31, 32, 35) showed moderate quality. Therefore, all articles were approved to be included in this systematic review considering the absence of articles with a high risk of biases (low quality).

## Discussion

### Immunogenicity of mRNA Vaccines

Messenger RNA-based vaccine is a new type of vaccine that has yet to be introduced for human implementation. COVID-19 is the first disease for which such vaccines will be approved for usage. Hence, the effectiveness of the vaccine in providing long-term protection against infection is not yet known. However, several clinical trials have been conducted to investigate its safety, efficacy and immunogenicity. To begin with, the results after administrating two doses have shown promising outcomes in healthy adults.

Regarding the immunogenicity, antibody responses in healthy infection-naïve individuals after immunisation presented in this study are comparable with the results obtained in clinical trials conducted by Jackson et al. (9), Anderson et al. (10), Walsh et al. (6), Widge et al. (11) and Chu et al. (12). The seronegative patient cohorts in these studies exhibited increased binding and neutralising antibodies against the spike protein following mRNA vaccination. Characteristically, after the first dose, the antibody responses in this group increased slightly from the baseline concentration and after the booster dose, the response increased remarkably. Essentially, these observations are common to those of other vaccines that require more than a single dose.

Mechanistically, the administration of mRNA vaccines leads to the presentation of spike antigens on antigen-presenting cells (APCs), first through major histocompatibility (MHC)-I and subsequently via MHC-II. Consequently, these antigens are carried by APCs to the lymph nodes, which leads to the priming of CD4 T cells (36). The differentiation of T helper 1 or T follicular helper cells by CD4 T cells initiates the formation of the germinal centre. Subsequently, specific memory B cells and long-lived plasma cells are produced at this site following the somatic hypermutation and positive selection processes. The generated plasma cells ultimately secrete binding and neutralising antibodies (37) which were detected in all studies included in this review.

Binding antibody eliminates infected cells through various pathways, such as the mechanism of antibody-dependent cell-mediated cytotoxicity (ADCC) associated with natural killer cells and antibody-dependent cell-mediated phagocytosis (ADCP) and complement-mediated cytotoxicity associated with phagocytes (38). All these pathways lead to the lysis and phagocytosis of infected cells. Meanwhile, the neutralising antibody interferes with the viral entrance and halts the infections (39, 40). Memory B cells are programmed to differentiate directly into plasma cells, and hence, specialised antibodies are observed during the actual infection or after the administration booster dose. Therefore, it is not uncommon for a minor antibody response after the primary dose and a sudden increase in response after the second dose in infection-naïve individuals. In conclusion, mRNA vaccines are adequately immunogenic to stimulate the production of antibodies that are essential for the removal of infected cells.

### Robust Antibody Response in Infected Individuals after Primary Dose

Individuals with a positive COVID-19 are distinctly characterised owing to their acquired immunity from natural infection. The acquisition starts from the entry of SARS-CoV-2 into host cells via ACE2 receptors, proceeding to the replication and multiplication of viral proteins. Subsequently, innate immunity is activated, which finally leads to the desired induction of adaptive immune cells. Specific antibodies, B cells and T cells for SARS-CoV-2 are produced during the infection (40).

The amount of antibodies declines after the convalescence phase (15, 41, 42). However, the immune memories of these cells are also retained for at least 6 months after the infection (43, 44). This is consistent with the results obtained from this study that showed the detectability of both binding and neutralising antibodies during the pre-vaccination phase in infected individuals. Most of the studies included in this systematic review examined participants that had COVID-19 infection 1 to 11 months (μ: 6.5 months) before the vaccination.

Consistent with the hypothesis, this study also presented the data of robust antibody responses in the same positive group after receiving the first vaccination, parallel to the outcomes observed in other vaccine-induced viral infections. For instance, a study conducted by Venkataswamy et al. (45) examining the immunogenicity of rabies vaccine and a study by Arciuolo et al. (46) investigating the immune response of measles, mumps and rubella (MMR) vaccine in post-exposure individuals showed similar results of strong antibody response after the immunisation. Correspondingly, antibody responses are more robust in symptomatic patients compared to asymptomatic patients (47, 48). This evidence justified the expected significant increase in antibody responses of pre-immunity groups after administration of primary vaccination dose due to the existing memory cells.

Theoretically, recall cells shorten the immune response time to re-encountering the same antigen produced by the vaccine and from the infection (49). Particularly, the immune system is capable of skipping the maturation and education processes during the primary immune responses (50). In consequence, direct activation of memory immune cells leads to quicker antibody production and hence, leads to high antibody titres in formerly infected individuals after the first vaccination. Thus, this generation of memory cells that do not need antigen stimulation to produce specialised spike protein antibodies is important for the prevention of severe relapse of the infection.

### Antibody Response in Infected Individuals after Booster Dose

Strong and high responses in infected individuals after the primary dose have been shown. On the contrary, only minor increases have been reported in antibody concentration after the second dose. The detailed mechanism or reason underlying observation remains unclear. Even so, these results may suggest close relation with the concept of steady-state phase in anamnestic response (i.e. secondary immune response) during which the antibody production is proportional to the antibody deterioration. Approximately, 90% of the antibodies are reported to degenerate within 90 days after the vaccination (51). Fundamentally, this steady-state phase is seen when the antibody concentration is not further increasing or merely plateauing in comparison to that observed after the previous dose.

However, small increments in response that were primarily observed in this study after the second dose may be explained due to the half-life of IgG antibodies that remained for at least a few weeks to 4 months (52) after production. The interval of 21 days between the first and second doses of mRNA vaccination may play a role in small increments in antibody concentration after the booster dose. Particularly, the mass production of IgG antibodies during the first dose in infected individuals inhibits (in time relation) antibody decline before the administration of the second dose. Therefore, only a small amount of antibodies is eliminated during the interval, and hence, only a small amount of antibodies could be produced. This mechanism has also been outlined in the study conducted by Wisnewski et al. (53) that examined the induction and decay of IgG antibodies produced in SARS-CoV-2 after mRNA vaccination. In summary, the slight increase in antibody concentration after the second dose in infected individuals is related to the balance of antibody production and deterioration. Above all, it can be inferred that antibody responses can be induced in previously infected individuals after receiving the first and second vaccination.

### Rapid Antibody Responses in Infected Individuals Compared to Those in Uninfected Individuals after mRNA Vaccination

As observed in the findings of articles included in this review, infected individuals undeniably demonstrate higher antibody titres compared to uninfected individuals at every vaccination time points. Specifically, during the baseline assessment, more anti-spike or anti-RBD titres are detected in previously infected individuals and little or none are spotted in uninfected individuals. Uninfected individuals are not yet confronted with the viruses, and hence, no immunity specific to the viral antigen has been activated. However, detectable antiserum levels at the baseline in the infection-naïve group may be attributed to the history of exposure to other coronaviruses, given that the SARS-CoV-2 antigen is highly similar to SARS-CoV and MERS-CoV antigens (54, 55). Thus, the traceability of SARS-CoV-2 antigen in the uninfected group during the pre-vaccination can be enhanced.

Furthermore, higher antibody response in seropositive individuals after the first dose can be attributed to the pre-existence memory cells. The primary dose in infected individuals acts as a trigger to induce robust antibody production (30), such as the anamnestic response. Meanwhile, in uninfected individuals, the first vaccination is the initiation of the primary immune response for which several days are needed for antibody production, which leads to a lower level of antibodies in seronegative groups. This observation is not only specific to COVID-19; it is also common in other viral infections, such as the measles studied by Anichini et al. (56).

Moreover, the pace or rapidity of achieving the highest antibody titres before plateauing is also one of the differences between previously infected and uninfected groups. Positive participants can reach higher antibody titres earlier (after the first dose) compared to the negative participants (after the second dose). Most of the results obtained in this study displayed the absence of substantial differences between the first and second doses of the infected group. It is important to note similar antibody titres observed after the first dose in infected individuals and after the second dose in uninfected individuals.

Mazzoni et al. (57) uncommonly showed a drop in antibody response in the infected group after the second dose. Levi et al. (31) also discussed the reduced serological response in formerly infected individuals that received the second dose compared to the same group that refused the booster dose. Furthermore, a higher reactogenicity is observed after second immunisation that may contribute to vaccine hesitancy in this pre-immunity group (24, 30, 58). These higher rates of side effects observed after the booster dose can be attributed to further increases in antibody production that leads to more cytokine secretion. Based on these observations, administration of second doses to previously infected individuals may lead to some detrimental effect. Despite this, both infected and uninfected individuals demonstrated an increase in binding and neutralising antibodies titres after mRNA vaccination. However, a more rapid increase in high antibodies concentrations is observed in infected individuals.

### Vaccination Practice for Infected Individuals

Based on the results, the infected individuals develop peak antibody responses one order of magnitude higher than the uninfected individuals. These results suggest that previously infected individuals should receive only one dose of mRNA vaccine. This strategy is similar to that presented in the study conducted by Van Buynder et al. (59) that proposed a single dose of influenza vaccine (typically administered in two doses) to be effective in preventing reinfection in children older than 10 years old with the previous history of H1N1. Hence, to pursue this vaccination regimen, several studies have suggested conducting serological tests before primary vaccination (28, 33, 59). These tests were conducted to identify uninfected and infected individuals to allocate single or double doses. This is beneficial to counter the limited supply of vaccines and difficulties in vaccine deployment that are currently being observed in many countries.

## Conclusion

mRNA vaccines induced robust binding and neutralising antibody responses in previously infected individuals, with similar results to those without history of COVID-19 infection. Principally, no significant differences were observed in the ability of the individuals in both groups to develop an immune system against the SARS-CoV-2. However, the infected individuals demonstrated higher antibodies concentrations at every vaccination time point. Furthermore, these formerly infected individuals also presented vigorous antibody responses right after the primary dose in contrast to infection-naïve individuals that require the booster dose. Correspondingly, a single dose of vaccination for the seropositive individuals should be sufficient to reach the same level of antibody concentration as observed in seronegative individuals after receiving two doses of vaccination. In spite of everything, it is important to be noted that longevity protection associated with these levels of titres in both infected and uninfected individuals is yet to be proven as studies on durability are still in progress.

### Limitations

Several limitations should be acknowledged in this study. Firstly, the specific focus on the humoral immune response of the vaccine, especially only on IgG antibodies, may not display a comprehensive view of the whole immune system. Other than that, most of the included studies involved healthcare workers. Therefore, the results obtained may not be extrapolated to determine immunogenicity outside of the clinical settings in other populations. Furthermore, several units were used in this study to express antibody concentrations, and hence, the direct comparison of concentration between the studies could not be performed.

### Future Study

Owing to the emergence of new viral genetic mutations in the near future, studies on the immunogenicity of the current and new variants are warranted. Furthermore, the durability of mRNA vaccines in protecting both uninfected and infected groups from infection and re-infection needs to be investigated. Other than that, a systematic review of cellular immunity response, such as that mediated by T lymphocytes, should also be performed, given that such a response is an important indicator of vaccine effectiveness.

## Figures and Tables

**Table 1:** Overview characteristics of included articles

Study	Author	Study design	Vaccine(s)	Participants (*n*)		Duration of infection before vaccination	Participant characteristics	Serological assay(s)

				Case (+)	Control (−)			
1.	Gobbi et al. (23)	Case-control study	BNT162b2	6	9	9 months	Healthcare workers, Italy	BAb: CMIA (IgG anti-RBD) NAb: MN assay
2.	Vicenti et al. (24)	Case-control study	BNT162b2	41	16	313 days	Healthcare workers, Italy	NAb: Live virus microassay
3.	Abu Jabal et al. (25)	Cohort study	BNT162b2	34	369	39 days–308 days	Healthcare workers, Israel	BAb: CLIA (IgG anti-spike)
4.	Ebinger et al. (26)	Cohort study	BNT162b2	35	228	Not stated	Healthcare workers, USA	BAb and surrogate NAb: CMIA (IgG-S-RBD)
5.	Padoan et al. (27)	Cohort study	BNT162b2	13	150	9 months	Healthcare workers, Italy	BAb: CLIA (IgG anti-spike and IgG anti-RBD)
6.	Goel et al. (28)	Cohort study	BNT162b2/mRNA-1273	11	33	65 days–275 days	Healthcare adults, USA	BAb: ELISA (IgG anti-spike and IgG-RBD) NAb: PsVNA
7.	Cavalcanti et al. (29)	Cohort study	BNT162b2	35	158	Not stated	Healthcare workers, Italy	BAb: ECLIA (IgG anti-RBD)
8.	Bayart et al. (30)	Cohort study	BNT162b2	72	159	99 days	Healthcare workers, Belgium	BAb: CMIA (IgG anti-S/RBD)
9.	Levi et al. (31)	Case-control study	BNT162b2	57	67	3 months	Healthcare workers, Italy	BAb: Indirect CLIA (IgG anti-S1/S2)
10.	Favresse et al. (32)	Case-control study	BNT162b2	73	158	34 days–337 days	Healthcare workers, Belgium	BAb: ECLIA (IgG anti-S)
11.	Salvagno et al. (33)	Cohort study	BNT162b2	206	719	Not stated	Healthcare workers, Italy	BAb: CLIA (IgG anti-RBD)
12.	Modenese et al. (34)	Case-control study	BNT162b2	31	43	3 months–11 months	Healthcare workers, Italy	NAb: ELISA (IgG-RBD)
13.	Kontou et al. (35)	Case-control study	BNT162b2	13	551	3 months–11 months	Healthcare workers, Greece	BAb: CMIA (IgG anti-S)

Notes: (+) individuals with history of COVID-19; (−) individuals without history of COVID-19; BAb = binding-antibody; NAb = neutralising antibody; IgG = immunoglobulin G; RBD = receptor-binding domain; S = spike; S1/S1 = subunit ½; CMIA = chemiluminescent microparticle immunoassay; MN = microneutralisation; CLIA = chemiluminescent immunoassay; ELISA = enzyme-linked immunosorbent assay; PsVNA = pseudovirion neutralisation assay; ECLIA = electrochemiluminescence immunoassay analyser

**Table 2:** Data on the binding and neutralising antibodies concentration in infected and uninfected individuals

Study	Author	Follow-up assessments	Antibody concentration (median)			

			Infected individuals		Uninfected individuals	
	
			Binding antibody	Neutralising antibody	Binding antibody	Neutralising antibody
1.	Gobbi et al. (23)	At baseline	497.6 AU/mL	325.5 (NT)	0.0 AU/mL	0.0 (NT)
		After first dose	64, 923.5 AU/mL	8,135.0 (NT)	7,992.5 AU/mL	252.0 (NT)
		After second dose	79, 546.0 AU/mL	10, 055.0 (NT)	78, 057.5 AU/mL	3,505.0 (NT)
2.	Vicenti et al. (24)	At baseline		26.0 (ID_50_)		< DV
		After first dose		1,544.0 (ID_50_)		5.0 (ID_50_)
		After second dose		N/A		183.0 (ID_50_)
3.	Abu Jabal et al. (25)	At baseline	68.6 AU/mL		< DV	
		After first dose	573.0 AU/mL		61.5 AU/mL	
4.	Ebinger et al. (26)	At baseline	6.0 AU/mL	7.7% a	< DV	0.0% a
		After first dose	10.0 AU/mL	77.1% a	7.0 AU/mL	7.6% a
		After second dose	N/A	100.0% a	9.9 AU/mL	97.4% a
5.	Padoan et al. (27)	At baseline	> 1.0 kAU/L b		< 1.0 kAU/L	
		After first dose	746.0 kAU/L		6.62.0 kAU/L	
		After second dose	1,713.0 kAU/L		382.0 kAU/L	
6.	Goel et al. (28)	At baseline	35.6 μg/mL	186.0 (FRNT_50_)	7.9 μg/mL	115.0 (FRNT_50_)
		After first dose	1,563.4 μg/mL	12, 219.9 (FRNT_50_)	269.5 μg/mL	285.6 (FRNT_50_)
		After second dose	2,027.2 μg/mL	18, 175.7 (FRNT_50_)	2,885.4 μg/mL	5, 819.0 (FRNT_50_)
7.	Cavalcanti et al. (29)	At baseline	36.6 BAU/mL		< 0.4 BAU/mL	
		After first dose	2500.0 BAU/mL		18.9 BAU/mL	
		After second dose	N/A		2,111.0 BAU/mL	
8.	Bayart et al. (30)	At baseline	299.0 AU/mL		< DV	
		After first dose	23, 515.0 AU/mL		370.0 AU/mL	
		After second dose	25, 508.0 AU/mL		15,591.0 AU/mL	
9.	Levi et al. (31)	At baseline	44.6 AU/mL		3.4 AU/mL	
		After first dose	1,182.8 AU/mL		52.9 AU/mL	
		After second dose	2,298.3 AU/mL		685.5 AU/mL	
10.	Favresse et al. (32)	At baseline	45.4 U/mL		< detectable value	
		After first dose	6,347.0 U/mL		34.4 U/mL	
		After second dose	11, 911.0 U/mL		1,312.0 U/mL	
11.	Salvagno et al. (33)	At baseline	68.0 U/mL		< 0.8 U/mL	
		After first dose	11, 782.0 U/mL		42.0 U/mL	
		After second dose	15, 142.0 U/mL		1,364.0 U/mL	
12.	Modenese et al. (34)	At baseline		35.2 BAU/mL		< DV
		After second dose		6,856.0 BAU/mL		3,746.0 BAU/mL
13.	Kontou et al. (35)	After first dose	2,118.2 AU/mL		568.5 AU/mL	
		After second dose	20, 547.0 AU/mL		10, 153.4 AU/mL	

Notes:

a proportion of participants surpassing 4,160 AU/mL (indicates the detectable NAb);

b indicates detectable/positive antibody; DV = detectable value; AU = arbitrary units; mL = millilitre; NT = neutralisation titre; ID_50_ = infectious dose 50%; N/A = not available; kAU = kilo arbitrary units; L = litre; μg = microgram; FRNT_50_ = focus reduction neutralisation titre 50%; BAU = binding antibody units; U = units

**Table 3:** Antibodies response of previously infected individuals according to vaccination timepoints

Study	Articles	Vaccination timepoint(s)	Main finding(s)*			

			Binding-antibody		Neutralising antibody	
	
			Concentration changes (%)	Interpretation	Concentration changes (%)	Interpretation
1.	Gobbi et al. (23)	At baseline	–	Detectable	–	Detectable
		After first dose	+99.23	Robust increase	+96.00	Robust increase
		After second dose	+18.41	Slight increase	+19.09	Slight increase
2.	Vicenti et al. (24)	At baseline			–	Detectable
		After first dose			+98.31	Robust increase
3.	Abu Jabal et al. (25)	At baseline	–	Detectable		
		After first dose	+88.03	Robust increase		
4.	Ebinger et al. (26)	At baseline	–	Detectable	–	Detectable
		After first dose	+40.00	Slight increase	+90.01	Robust increase
		After second dose			+22.90	Slight increase
5.	Padoan et al. (27)	At baseline	–	Detectable		
		After first dose	+99.73	Robust increase		
		After second dose	+56.45	Slight increase		
6.	Goel et al. (28)	At baseline	–	Detectable	–	Detectable
		After first dose	+97.72	Robust increase	+98.48	Robust increase
		After second dose	+22.88	Slight increase	+32.77	Slight increase
7.	Cavalcanti et al. (29)	At baseline	–	Detectable		
		After first dose	+98.54	Robust increase		
8.	Bayart et al. (30)	At baseline	–	Detectable		
		After first dose	+98.73	Robust increase		
		After second dose	+7.81	No changes		
9.	Levi et al. (31)	At baseline	–	Detectable		
		After first dose	+96.23	Robust increase		
		After second dose	+48.54	Slight increase		
10.	Favresse et al. (32)	At baseline	–	Detectable		
		After first dose	+99.28	Robust increase		
		After second dose	+46.71	Slight increase		
11.	Salvagno et al. (33)	At baseline	–	Detectable		
		After first dose	+99.42	Robust increase		
		After second dose	+22.19	Slight increase		
12.	Modenese et al. (34)	At baseline			–	Detectable
		After second dose			+99.49%	Robust increase
13.	Kontou et al. (35)	After first dose	–	Robust increase		
		After second dose	+89.69	Robust increase		

Note:

* Based on the data in Table 2

**Table 4:** Result of risk of bias assessments of retrieved articles based on the CCAT

Study	Articles	Categories								Total score

		Preliminaries	Introduction	Design	Sampling	Data collection	Ethical matters	Results	Discussion	
1.	Gobbi et al. (23)	4	4	3	4	4	3	4	5	31
2.	Vicenti et al. (24)	3	4	4	4	3	3	3	4	28
3.	Abu Jabal et al. (25)	4	5	4	4	4	3	4	4	32
4.	Ebinger et al. (26)	4	5	4	4	4	4	4	4	33
5.	Padoan et al. (27)	5	5	3	4	5	3	4	4	33
6.	Goel et al. (28)	5	5	4	3	5	3	3	4	32
7.	Cavalcanti et al. (29)	4	5	3	4	3	3	3	3	28
8.	Bayart et al. (30)	4	5	3	4	3	4	4	5	32
9.	Levi et al. (31)	4	4	3	3	4	4	4	3	29
10.	Favresse et al. (32)	3	3	3	4	4	3	3	3	26
11.	Salvagno et al. (33)	4	5	4	5	5	5	4	4	36
12.	Modenese et al. (34)	4	5	4	4	4	5	4	3	33
13.	Kontou et al. (35)	3	3	3	3	3	5	4	5	29

**Table 5:** Total results of risk of bias assessments of retrieved articles

Study	Articles	Percentage score	Quality
1.	Gobbi et al. (23)	78	High
2.	Vicenti et al. (24)	70	Moderate
3.	Abu Jabal et al. (25)	80	High
4.	Ebinger et al. (26)	83	High
5.	Padoan et al. (27)	83	High
6.	Goel et al. (28)	80	High
7.	Cavalcanti et al. (29)	70	Moderate
8.	Bayart et al. (30)	80	High
9.	Levi et al. (31)	73	Moderate
10.	Favresse et al. (32)	65	Moderate
11.	Salvagno et al. (33)	90	High
12.	Modenese et al. (34)	83	High
13.	Kontou et al. (35)	73	Moderate
